# Predicting as a learning strategy

**DOI:** 10.3758/s13423-021-01904-1

**Published:** 2021-03-25

**Authors:** Garvin Brod

**Affiliations:** 1 DIPF | Leibniz Institute for Research and Information in Education, 60323 Frankfurt, Germany; 2 Department of Psychology, Goethe University, Frankfurt, Germany

**Keywords:** Learning techniques, Guessing, Generating predictions, Testing effect, Errorful learning, Retrieval practice

## Abstract

This article attempts to delineate the procedural and mechanistic characteristics of predicting as a learning strategy. While asking students to generate a prediction before presenting the correct answer has long been a popular learning strategy, the exact mechanisms by which it improves learning are only beginning to be unraveled. Moreover, predicting shares many features with other retrieval-based learning strategies (e.g., practice testing, pretesting, guessing), which begs the question of whether there is more to it than getting students to engage in active retrieval. I argue that active retrieval as such does not suffice to explain beneficial effects of predicting. Rather, the effectiveness of predicting is also linked to changes in the way the ensuing feedback is processed. Initial evidence suggests that predicting boosts surprise about unexpected answers, which leads to enhanced attention to the correct answer and strengthens its encoding. I propose that it is this affective aspect of predicting that sets it apart from other retrieval-based learning strategies, particularly from guessing. Predicting should thus be considered as a learning strategy in its own right. Studying its unique effects on student learning promises to bring together research on formal models of learning from prediction error, epistemic emotions, and instructional design.

Asking students to generate a prediction before presenting the correct answer is a popular instructional strategy. It comes in many forms. For example, language teachers ask students to predict what comes next in a text (Fielding et al., 1990; Palinscar & Brown, 1984); science teachers ask students to predict the outcome of an experiment before conducting it (Inagaki & Hatano, 1977; Liew & Treagust, 1995); likewise, university lecturers use digital devices to poll their students for predictions before explaining a new concept (Crouch et al., 2004; Miller et al., 2013). In all of the cited studies, a beneficial effect of letting students generate predictions on learning outcomes was reported. But what are the exact mechanisms by which predicting leads to better learning? Do they go beyond the well-known benefits of active retrieval? And does it make a difference if learners generate an informed prediction or if they quickly guess an answer?

This article attempts to delineate the procedural and mechanistic characteristics of predicting as a learning strategy. While procedural characteristics refer to how a strategy is commonly implemented, mechanistic characteristics refer to the cognitive processes that this strategy taps into. As is apparent from the examples above, asking learners to predict outcomes or answers prompts them to go beyond the provided information. Doing so requires active retrieval of semantic knowledge, which is the mechanistic hallmark of generative learning strategies (Brod, 2020). Predicting thus belongs to the group of generative learning strategies (Fiorella & Mayer, 2016; Wittrock, 2010). Within this group, predicting shares many procedural characteristics with other strategies, including practice testing, pretesting, and guessing. Collectively, I will call this subgroup retrieval-based learning strategies because they entail that learners have to attempt to retrieve information from memory in order to generate a response, which is typically followed by presentation of the correct answer (S. H. K. Kang et al., 2011; Karpicke & Roediger, 2008; Kornell et al., 2009; Potts & Shanks, 2014). The main objective of the current article is a taxonomical one; I want to show that predicting constitutes a learning strategy in its own right within the group of retrieval-based learning strategies. To this end, I will compare the procedural and mechanistic characteristics of predicting with those of other prominent generative learning strategies that capitalize on active retrieval (i.e., guessing, pretesting, and practice testing). A rough summary of those characteristics as well as of differences and commonalities between strategies is provided in Table 1.

**Table 1:** Differences and commonalities between different retrieval-based learning strategies

	Guessing	Predicting	Pretesting	Testing
Procedural characteristics	Learners generate a response with little confidence, followed by immediate feedback.	Learners generate a response with at least some confidence, followed by immediate feedback.	Learners typically generate several responses first, study opportunities/feedback follow later.	Learners study material first, followed by memory tests with/without feedback
Mechanistic characteristics	Semantic elaboration Increased attention to feedback	Semantic elaboration Increased attention to feedback Surprise (if incorrect)	Semantic elaboration Increased attention to feedback	Semantic elaboration Additional episodic context cues
Exemplary studies	Kornell et al., 2009; Potts & Shanks, 2014	Brod et al., 2018; Miller et al., 2013	Kornell, 2014; Richland et al., 2009	Carpenter & DeLosh, 2005; Karpicke & Roediger, 2008

I will address the procedural and mechanistic level successively, beginning with the question of how the different strategies are commonly implemented. This is followed by an investigation of whether predicting evokes additional mechanisms beyond active retrieval. In short, it is found that a specific element of predicting is that it boosts learners’ surprise about unexpected outcomes, which leads to enhanced attention and facilitates encoding of the correct answer. The article closes by providing directions for future research that brings together formal models of learning from prediction error, epistemic emotions, and instructional design.

This article only considers predicting as a strategy for simple declarative learning (e.g., facts, definitions). It does not include predicting as a strategy for inducing complex conceptual change, for which it is popular as well (e.g., Champagne et al., 1982; Hardy et al., 2006). It is also restricted to direct, task-specific mnemonic effects of predicting. Plausible additional indirect effects such as that predicting helps students to identify gaps in their knowledge or that it raises their motivation to study are thus ignored.

## Procedural commonalities and differences between predicting and other retrieval-based learning strategies

To delineate predicting from other retrieval-based learning strategies, it is crucial to first examine how these strategies are commonly implemented because they also come in many forms. The most straightforward form of practice testing (also called retrieval practice) involves retrieval of information that was learned shortly before, such as retrieving names corresponding to faces, without any kind of feedback regarding correctness of retrieval. This basic type of practice testing already improves retention more than restudying does, indicating a direct effect of active retrieval on memory (e.g., Carpenter & DeLosh, 2005). What is more common, however, is practice testing with feedback, mostly in the form of several restudy opportunities. Practice testing with feedback has been shown to be more effective than testing alone (Butler & Roediger, 2008). In sum, whether or not practice testing includes feedback, it always includes an initial study phase followed by one or more retrieval phase(s) in which learners have to retrieve parts of the material presented in the study phase (i.e., intentional retrieval from episodic memory; see Karpicke & Zaromb, 2010; Mulligan et al., 2016). Practice testing is thus clearly different from predicting, which does not include an initial study phase.

Studies that have examined errorful generation have used tasks that are more similar to predicting. In this approach, learners are prompted to generate answers before they have gone through a study phase or even before they have been exposed to the relevant material, which means that learners commit a lot of errors (i.e., *errorful* generation). This approach has been successfully implemented in studies on *pretesting* that let learners answer questions well before the correct answers are revealed, such as in a text that learners have to read later on (Kornell, 2014; Richland et al., 2009). More frequently, learners are asked to *guess* immediately before the correct answer is revealed. For example, learners are asked to guess translations of foreign language vocabulary before they are shown the correct translations (Potts & Shanks, 2014), or they have to answer fictitious general-knowledge questions before they are shown the correct answers (Kornell et al., 2009). In all of these implementations, learners are hardly ever able to retrieve the correct answer. Nevertheless, the retrieval attempt alone can promote learning of the correct answer (Potts et al., 2019; Seabrooke et al., 2021). A boundary condition for the benefit of guessing to occur is that the correct answer has to be presented shortly after the guess (Vaughn & Rawson, 2012). In sum, errorful generation can be considered a special case of the generation effect (Bertsch et al., 2007; Slamecka & Graf, 1978), which denotes that actively produced information is better remembered than information that is just read.

Is there a difference between guessing followed by a study opportunity and predicting? Indeed, predicting and guessing have sometimes been used interchangeably in articles on retrieval-based learning (Brod & Breitwieser, 2019; Yan et al., 2014; Zawadzka & Hanczakowski, 2018), but they often carry different connotations. Guessing as a learning strategy can be defined as prompting learners to generate “a response to hitherto unfamiliar materials” (Potts et al., 2019), which implies a lack of preexisting semantic associations.^1^ This is different for predicting as delineated here, which involves at least some familiarity with the materials and thus evokes preexisting semantic associations (Brod et al., 2018; Cyr & Anderson, 2018). Familiarity with the materials allows learners to commit to one (out of several possible) outcomes with at least some confidence (Brod et al., 2017). In sum, predicting could be distinguished from guessing by the higher degree of confidence that learners place in their response.

It is obvious that drawing a line between guessing and predicting based on overt procedural characteristics can be difficult, particularly for studies that did not assess learners’ confidence. Therefore, in the following section, in which I compare the mechanistic characteristics of predicting with those of the other retrieval-based learning strategies, a pragmatic distinction is drawn between predicting and guessing. I use the term *predicting* for studies that analyzed both correct and incorrect initial responses. Guessing is used for those studies that excluded correct responses (which were also few in number), which suggests that participants were expected to be unfamiliar with the materials.

## Mechanistic commonalities and differences between predicting and other retrieval-based learning strategies

Semantic elaboration has arguably been the most prominent mechanism suggested to underlie the benefits of attempting to retrieve items from memory. Semantic elaboration could benefit later retrieval because it provides additional links between the cue and the target (Kornell, 2014; Pyc & Rawson, 2010, 2012). For the example of vocabulary learning, this would mean that in the search process for the correct translation, semantically related items are retrieved as well and encoded alongside the foreign word and its translation. These semantically related words then serve as mediators that provide additional pathways from the word to the correct translation, which increases the probability of future retrieval success. If the retrieved translation is incorrect, the incorrect translation itself can serve as a mediator between the foreign word and the correct translation (Huelser & Metcalfe, 2012; Vaughn & Rawson, 2012). In addition, the feedback that the translation was incorrect together with the presentation of the correct translation could prompt learners to shift to better mediators when faced with the same word later on (Pyc & Rawson, 2012). Attempting to retrieve items from memory could, thus, benefit later retrieval because it generates semantic mediators, independent of whether the initial retrieval attempt was successful. Alternatively or additionally, spreading activation theory suggests that if the correct item gets co-activated during the retrieval attempt, this semantic preactivation could improve encoding of the item when it is revealed (Carpenter, 2009; Zawadzka & Hanczakowski, 2018). Semantic elaboration processes triggered by a retrieval attempt could thus explain the benefits of all retrieval-based learning strategies.

An alternative, episodic context account of active retrieval suggests that repeated retrieval of an item in multiple contexts leads to an increase in the number of retrieval cues (i.e., the different contextual features), which likewise increases probability of future retrieval success (Karpicke et al., 2014). This account was put forward to explain the benefits of practice testing with multiple retrieval phases. It is therefore not directly applicable to explaining the benefits of predicting, pretesting, or guessing, which are typically performed only once for a particular cue–target combination. I will, therefore, focus on the semantic elaboration account, which could explain both the benefits of practice testing and errorful generation (see, e.g., Kornell et al., 2009; Zawadzka & Hanczakowski, 2018).

## Can the benefits of predicting be explained by semantic elaboration alone?

The benefits of predicting can at least in parts be explained by the semantic elaboration account as well. A recent study has elegantly demonstrated incorrect predictions being particularly helpful when they semantically overlap with the correct answer (Cyr & Anderson, 2018). English-speaking students were asked to predict the English translation of Spanish words before seeing the correct translation. The Spanish words were chosen so that they resemble an English word but mean something different, thus leading to many incorrect predictions (correct predictions were not excluded, however). The key manipulation was that some of the Spanish words were semantically distant from the English word (e.g., *carpeta*–*folder*, mismatch condition), whereas others were semantically close and comparably easy to integrate semantically (e.g., *carrera*–*degree*, match condition). Recall performance was compared with a study condition in which both the Spanish word and the correct English translation were presented immediately. Predicting benefitted recall in the match condition only. This finding was taken to suggest that mostly incorrect albeit semantically close predictions could serve as better mediators during later retrieval than semantically distant predictions.

The benefits of predicting for later recall go beyond the explanatory realm of the semantic elaboration account of retrieval practice, however. This has recently been demonstrated in a series of experiments in which participants were presented with a cue word and had to predict a related target word (Zawadzka & Hanczakowski, 2018). Of note, the cue word was a homograph with two different meanings (e.g., *ball*), which yielded a similar number of predictions that were correct or semantically related (e.g., *goal*) and predictions that were semantically distant to the intended meaning of the cue word (e.g., *mask*). As in Cyr and Anderson’s (2018) study, predicting was advantageous relative to studying when the predictions matched the intended meaning of the cue word, thus providing support for the semantic elaboration account. However, when cued recall was prompted using a word that was different than the cue but related to both the cue and the target (e.g., *dress*), there was a benefit of predicting relative to studying for semantically distant predictions as well, which was equal in size to the one for semantically related predictions. This result is in conflict with the semantic elaboration account, which assumes that links between the original cue, the mediator, and the target are forged only during generation. Rather, the findings suggest that even a prediction that is semantically distant from the target can improve its retention if it improves processing of the correct answer later on.

Zawadzka and Hanczakowski (2018) speculate that enhanced attention to the presentation of the correct answer, triggered by resolving of curiosity as well as surprise, might explain the observed benefit for semantically distant predictions. Recently, several studies have indicated that enhanced attention to the correct answer might indeed constitute a second mechanism underlying the benefits of predicting. These studies will be discussed in the next section.

### Predicting stimulates curiosity

Curiosity has been suggested to arise from a perceived knowledge gap (Loewenstein, 1994) and to involve an increase in arousal, which results in increased attention and improved memory encoding (Berlyne, 1954). Surprise is thought to arise from a violation of expectation and to likewise involve a temporary increase in arousal that has motivational and cognitive effects such as increased attention (Reisenzein et al., 2019). Both curiosity and surprise are thus assumed to initiate cognitive processing in the service of learning, hence both have been classified as *epistemic emotions* (e.g., Pekrun et al., 2017).

Recently, Brod and Breitwieser (2019) examined the effects of predicting on curiosity in a numerical trivia facts task (e.g., “How many out of 10 birds are migratory birds?”). Participants either had to predict the answer or generate an example of the fact (e.g., “wild goose”), followed by a curiosity rating and feedback on the correct answer. It was found that generating predictions led to higher curiosity ratings than generating examples, indicating that generating predictions indeed triggers curiosity. While higher curiosity ratings were associated with better memory for the fact, the two conditions did not differ in overall retention rates. Of note, task-related pupil dilations were also studied as a measure of the noradrenergic modulation of arousal, and thereby attention, which should be enhanced for high-curiosity facts. Indeed, during anticipation of the answer, high-curiosity facts were associated with a larger pupil dilation than were low-curiosity facts in the prediction condition. However, this effect did not extend to the feedback phase in which participants saw the correct answer, for which there was no difference in pupil dilation as a function of curiosity. Together, these findings indicate that while prediction-induced curiosity results in greater attention during the generation of the prediction, it may not lead to enhanced attention to the correct answer later on. It is thus unclear whether the increased curiosity facilitates encoding of the correct answer or merely of the (mostly incorrect) prediction.

Does increased curiosity contribute to the beneficial effects of guessing as well? The picture is currently unclear. Curiosity has been shown to follow an inverted-U-shaped function of confidence in the prediction (e.g., Kang et al., 2009). Guessing is thus rather unlikely to yield a high degree of curiosity. Nevertheless, a recent study found higher curiosity ratings when participants guessed before the rating than when they guessed after the rating (Potts et al., 2019). The two conditions did not lead to differences in memory performance, however, and higher curiosity was not associated with better memory. These findings are similar to those on predicting (Brod & Breitwieser, 2019), for which the relation between curiosity and later memory remained unclear as well. It is, thus, unclear whether increased curiosity contributes to the beneficial effects of guessing.

To conclude, while both predicting and guessing might stimulate curiosity, it is currently unclear whether curiosity leads to enhanced attention only during the generation phase or also in response to the answer. How could generating predictions enhance attention to the feedback, particularly when the prediction was incorrect? It has been proposed that generating predictions leads to greater surprise when the prediction was incorrect, which in turn results in enhanced attention and better encoding of the correct answer (Brod et al., 2018). Direct and indirect evidence for this claim will be reviewed in the following sections.

### Predicting boosts surprise

Indirect evidence for a beneficial role of surprise in learning from feedback can be drawn from research on the *hypercorrection effect*. The hypercorrection effect refers to the finding that erroneous responses committed with high confidence are more likely to be corrected than erroneous responses committed with low confidence (Butterfield & Metcalfe, 2001). In their original experiment, Butterfield and Metcalfe (2001) asked students to predict answers to trivia questions, followed by confidence ratings and presentation of the correct answer. In a retest 5 minutes later, participants demonstrated better memory for those correct answers that they had initially predicted incorrectly with high confidence than with low confidence. The authors suggested that this effect is due to the corrective feedback being more surprising for high-confidence errors than for low-confidence errors, and that the ensuing increased arousal facilitates encoding of the correct answer. Subsequent studies provided evidence for this suggestion by demonstrating that electrophysiological markers of surprise and increased arousal during the presentation of the correct answer were associated with correct retention later on, in particular for those answers that were predicted incorrectly with high confidence (Butterfield & Mangels, 2003). Further evidence for this account was provided in a study that tested both memory for the correct answer and memory for irrelevant surface features of this answer, such as its font color (Fazio & Marsh, 2009). It was found that incorrect predictions made with high confidence improved memory for both, which suggests increased attention during the feedback presentation.

While findings on the hypercorrection effect lend support to the idea that surprising outcomes lead to enhanced attention, which in turn leads to facilitated memory encoding, they cannot substantiate whether this effect is because participants generate a prediction beforehand. The studies did not include a control condition in which participants did not generate an answer prior to the feedback. It is thus possible that the hypercorrection effect could be observed as well if participants indicated their prior belief as well as their confidence in this belief after having seen the correct answer. The causal role of predicting in driving the improved memory for surprising answers is thus unclear.

**Fig. 1:**
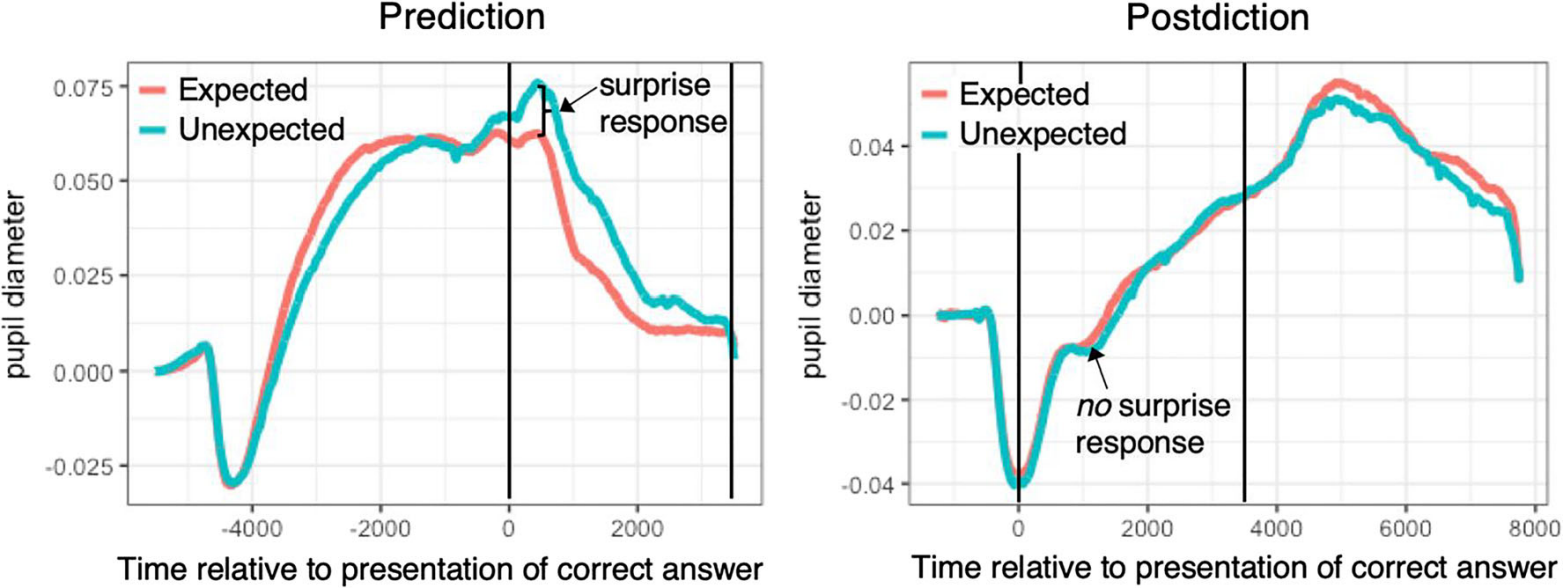
Generating a prediction triggers surprise. Depicted is the full time series of the pupillary response in the prediction (left) and postdiction condition (right), separately for expected and unexpected outcomes. There was a clear surprise response (i.e., positive difference in pupil diameter between unexpected and expected outcomes) to the presentation of the correct outcome (0 ms) in the prediction condition. There was no difference in pupil dilation in the postdiction condition. Black lines indicate the time during which the correct outcome was presented (figure based on Brod et al., 2018)

In two experiments, we tested the causal role of predicting with regard to boosting surprise and memory for the correct answer (Brod et al., 2018). Predicting was compared with a condition in which participants stated their predictions afterwards (*postdiction* condition). The only difference between conditions was the presentation order of the stimuli. In the first experiment, we found that incorrectly predicted outcomes in a geography knowledge task induced a succinct pupil dilation to the presentation of the correct answer (see Fig. 1), which is indicative of surprise and enhanced attention to the feedback. This was not the case in the postdiction condition, which indicates that generating a prediction beforehand boosted surprise about the correct answer. The size of the pupil dilation in response to an incorrectly predicted answer was further positively related to the correction of this prediction in a later test. A second experiment confirmed the beneficial effect of predicting on memory. Here, participants had to predict or postdict results of soccer matches. It was found that particularly incorrectly predicted outcomes were better remembered in the prediction condition than in the postdiction condition, indicating facilitated encoding due to enhanced surprise. In sum, findings indicate that predicting boosts both surprise and memory for the correct answer.

A follow-up study confirmed that it is not the case that any generative activity before presentation of the correct answer suffices to induce surprise (Breitwieser & Brod, 2021). Here, generating predictions was compared with generating examples. It was found that only generating predictions induced a pupil dilation response to the presentation of the correct answer. This pupil dilation response was further associated with later retrieval success. These findings thus provide further support for the claim that surprise induced by incorrect predictions facilitates encoding of the correct answer.

Does guessing suffice to induce surprise? The degree of surprise about an unexpected answer is a function of learners’ confidence in their erroneous belief, which has been demonstrated both using formal models of surprise (Itti & Baldi, 2009) and empirically for trivia facts questions (Reisenzein, 2000). This means that incorrect guesses, which are by definition provided with low confidence, are highly unlikely to elicit strong surprise reactions. This indicates that surprise cannot be a major driving force underlying the effectiveness of guessing. In line with this theoretical argument, a recent study that let participants rate their surprise after guessing found no difference in surprise ratings between guess trials and study trials (Seabrooke et al., 2019). In sum, both empirical and theoretical evidence suggest that guessing does not elicit strong surprise reactions for unexpected outcomes or answers. Surprise thus qualifies as a mechanism that distinguishes between the learning strategies of guessing and predicting.

The mechanistic role of surprise in boosting learning has been elucidated in recent cognitive neuroscience studies. Surprise as measured by pupil dilations is closely coupled with the release of norepinephrine in the brainstem’s locus coeruleus (Joshi et al., 2016). Release of norepinephrine by the locus coeruleus influences arousal levels and has been shown to prioritize processing of goal-relevant information and to promote memory formation in the hippocampus (Clewett et al., 2018; Mather et al., 2016). Applying these findings to the current goal of describing the cognitive mechanisms of predicting, a causal chain has been proposed in which (1) incorrect predictions lead to surprise, (2) surprise leads to enhanced attention to the correct answer, and (3) enhanced attention to the correct answer strengthens its encoding (Brod et al., 2018).

## Directions for future research

In the previous section, I attempted to delineate predicting as a learning strategy by comparing its mechanisms with those of other retrieval-based learning strategies. It became clear that the beneficial effects of predicting are not sufficiently explained by semantic elaboration alone, which is the key mechanism suggested for the other strategies. Rather, its effectiveness is also linked to changes in the way the ensuing information is processed. A growing body of evidence suggests that predicting boosts surprise about unexpected answers, which leads to enhanced attention and consequently to better encoding of the correct answer. Surprise thus qualifies as a mechanism that sets predicting apart from other retrieval-based learning strategies. It is currently unclear whether there are additional mechanisms (e.g., curiosity) that set predicting apart, and whether they could boost memory for expected outcomes as well. In this final section, I will argue that considering predicting as a learning strategy in its own right also opens up new vistas for future research.

Predicting already forms part of science teaching curricula, such as in the widely used “predict–observe–explain” curriculum (Champagne et al., 1982; Liew & Treagust, 1995). The psychological mechanisms by which these curricula work have remained opaque, however. Therefore, maximizing surprise in learners has not been a major target in teaching curricula thus far, but the findings summarized here indicate that it helps to raise learners’ attention on the outcome. Science education seems an ideal test case because its goal is to correct scientific misconceptions, which indicates that most predictions will be incorrect and surprise will be high. A natural follow-up question is how to best leverage learners’ attention. A prerequisite for doing so is to test whether predicting outcomes based on a complex theory differs from predicting isolated facts. Is it only the amount of memory retrieval that differs or does it also affect the way in which the feedback is processed? Knowledge of the mechanisms by which predicting promotes science learning can help to make these curricula more effective.

Furthermore, predicting as an instructional strategy touches on a highly topical research field in computational cognitive science that deals with the effects of prediction errors. Prediction errors play a key role in knowledge restructuring throughout the human brain (Bar, 2007). They have long been known to drive associative learning, which resulted in well-established formal models of learning from prediction error (Glimcher, 2011; Rescorla & Wagner, 1972). Recent research indicates that they facilitate declarative memory encoding as well (Greve et al., 2017). Note that, even though these formal models of prediction error are sometimes proposed to reflect surprise, they do not take into account the physiological experience of surprise in human learners. It is thus not necessarily the case that, even under ideal circumstances, there is a one-to-one correspondence between measures of surprise intensity and formal models of prediction error. It will nevertheless be fruitful to test whether the formal models developed to explain the effects of prediction errors in associative learning can explain the effects of predicting in educational scenarios as well.

Finally, predicting provides an ideal test case for studying the impact of emotions on learning, which has recently gained considerable traction (Sinatra et al., 2014). As discussed above, predicting has repeatedly been shown to reliably induce both curiosity and surprise, which can be classified as epistemic emotions (Pekrun et al., 2017). This firm link opens up the possibility to examine the causal chain from epistemic emotions to learning because it provides way to quasi-experimentally induce curiosity or surprise in students. Predicting could serve as a tool to study the interplay between epistemic emotions and learning, ideally using the formal modeling approaches mentioned above. This is made possible by recent developments in intelligent tutoring systems that use eye-tracking to capture emotional states for adapting instruction (e.g., D’Mello et al., 2012). This line of research promises to contribute both to improving student learning and to the formulation of stronger theories regarding the mechanisms by which emotions shape memory formation.

## Conclusions

All in all, I propose that predicting should be considered as a learning strategy in its own right. I argue that both its procedural and mechanistic characteristics differ from other retrieval-based learning strategies. Predicting involves that learners generate a response with at least some confidence, followed by immediate feedback. Similar to other retrieval-based learning strategies, predicting derives parts of its effectiveness from leading learners to retrieve information from memory and engage in semantic elaboration. However, incorrect predictions additionally evoke epistemic emotions, particularly surprise at seeing the correct answer. It could be demonstrated that the surprise response for incorrectly predicted outcomes is associated with later retrieval success over and above semantic elaboration. This specific effect of predicting sets it apart from other retrieval-based learning strategies, particularly from guessing. It needs to be noted, however, that the differences between guessing and predicting are largely differences in degree. At what point continuous differences, even if they exist on multiple characteristics, give rise to categorical differences is subject to debate. This review has argued that considering predicting as a learning strategy in its own right can promote research that brings together formal models of learning from prediction error and surprise with the design of instructional curricula. Knowledge of the precise mechanisms by which predicting promotes learning will contribute to making these curricula more effective.

## Footnotes

1 Note that, apart from guessing as a learning strategy, the term is also used in a broader sense to indicate that participants have very little confidence in their response, independent of whether the materials are familiar or unfamiliar (e.g., Butler & Roediger, 2008; S. H. K. Kang et al., 2011).
